# Quality of breast reconstruction service at a university hospital as assessed by the patients

**DOI:** 10.1590/acb381223

**Published:** 2023-05-01

**Authors:** Carlos Eduardo de Oliveira, José da Conceição Carvalho, Ricardo Beckhauser Kuhnen, Ana Laura Batista Coelho, Isabella Scavariello Zicari Di Monte, Lydia Masako Ferreira, Daniela Francescato Veiga

**Affiliations:** 1 Universidade Federal de São Paulo – Programa de Pós-graduação em Cirurgia Translacional – São Paulo (SP), Brazil.; 2 Universidade do Vale do Sapucaí – Faculdade de Medicina – Pouso Alegre (MG), Brazil.; 3 Universidade de Santo Amaro – Faculdade de Medicina – Santo Amaro (SP), Brazil.; 4 Universidade do Vale do Sapucaí – Divisão de Cirurgia Plástica – Pouso Alegre (MG), Brazil.

**Keywords:** Breast Neoplasms, Mammaplasty, Patient Satisfaction, Health Services Administration

## Abstract

**Purpose:**

To evaluate the quality of breast reconstruction service at a university hospital, as assessed by the patients.

**Methods:**

This cross-sectional study enrolled adult women who underwent immediate or delayed breast reconstruction by any technique performed at a university hospital between 1 and 24 months before the assessment. The Brazilian version of the Health Service Quality Scale (HSQS) was self-applied to the participants. The HSQS produces percentage scores, which are expressed in values ranging from 0 to 10 for each domain of the scale, and into an overall percentage quality score. The management team was asked to establish a minimum satisfactory score for the breast reconstruction service.

**Results:**

Ninety patients were included. The management team considered 8.00 the minimum satisfactory score for the service. The overall percentage score was 93.3%. Only one domain, ‘Support,’ had an average score lower than that considered satisfactory (7.22 ± 3.0); while the others had higher scores. The domain that scored highest was ‘Qualification’ (9.94 ± 0.3), followed by ‘Result’ (9.86 ± 0.4). There was a positive correlation between ‘type of oncologic surgery’ and ‘intentions of loyalty to the service’ (ρ = 0.272; p = 0.009) and a negative correlation between ‘education’ and ‘quality of the environment’ (ρ = –0.218; p = 0.039). The higher the patient’s level of education, the higher the score attributed to ‘relationship’ (ρ = 0.261; p = 0.013) and the lower the score of ‘aesthetics and functionality’ (ρ = –0.237; p = 0.024).

**Conclusions:**

The quality of the breast reconstruction service was considered satisfactory, but there is a demand for structural improvements, better interpersonal relationships, and a stronger support network for patients.

## Introduction

The incidence of breast cancer is high. In Brazil, excluding nonmelanoma skin tumors, breast cancer is the most frequent in women in all regions, with higher rates in the South and Southeast regions. For 2023, 73,610 new cases are estimated, which represents an adjusted incidence rate of 41.89 cases per 100,000 women^1^. Some studies have shown the impact of breast reconstruction on breast cancer patients’ quality of life, positively influencing psychosocial factors, sexuality, and general functioning^2^^-^ 4. Patient-centered care is particularly important in this kind of procedure, whose primary aim is to improve appearance and quality of life. Therefore, focusing on patient perceptions offers valuable opportunities to improve the effectiveness of healthcare^5^.

There is a consensus between health services providers and managers that quality of service and customer satisfaction are important strategic goals. Quality measurement is not only a technical issue, but has also implications for the allocation of health resources and the organization of medical practice^6^. Most of the Brazilian population is assisted in public or university hospitals linked to the Unified Health System, where the distribution of available resources is always a challenge.

Therefore, it is mandatory to identify opportunities to improve the services provided to maximize patients’ satisfaction and well-being. However, the literature on the quality of health services is scarce, especially in Brazil. Studies on the quality of Brazilian university health services were not found in the current literature.

The Health Service Quality Scale (HSQS) is a validated multidimensional hierarchical scale used to assess the quality of interpersonal relationships, technical quality, and the environmental and administrative quality of health services^6^. It has been translated into Brazilian Portuguese and validated for use in Brazil^7^. This study aimed to evaluate the quality of breast reconstruction service at a university hospital as assessed by the patients. Thus, the main objective of this study is to draw attention to the importance of this topic and its applicability in improving the quality of health services.

## Methods

This cross-sectional observational study was carried out at a university hospital from January to December 2019. The study was approved by the institutional Ethics Committees (CAAEE No. 00522918.0.0000.5505 and No. 42564815.0.0000.5102), and all patients provided written consent to participate.

Women aged over 18 years who underwent immediate or delayed breast reconstruction by any technique (implant-based or autologous reconstruction) at the university hospital between 1 and 24 months before the assessment were eligible. Women who had undergone breast reconstruction for causes other than oncologic surgical repair (e.g., trauma, Poland syndrome), who were illiterate, or who had a physical disability that prevented them from reading were excluded. Women who gave up completing the instrument or who withdrew their consent after completing it were considered losses and were not included in the data analysis. Patient’s main clinical and demographic characteristics were recorded.

A validated instrument, the Brazilian version of the HSQS, was self-applied in an outpatient return visit^6^^,^ 7. The HSQS comprises 73 statements, which are grouped into 16 domains with numerical values ranging from 1 to 7, corresponding to responses that range from ‘totally disagree’ to ‘completely agree.’ The numerical values are transformed into percentage scores, which are expressed in values ranging from 0 to 10 for each domain, and an overall percentage quality score.^6^^,^ 7

The endpoints were patients’ perceptions regarding the HSQS domains: Quality assigned to the service, Satisfaction with the service, Intentions of loyalty to the service, Quality interpersonal relationships, Technical quality, Environment quality, Administrative quality, Interaction, Relationship, Result, Qualification, Environment, Aesthetics and functionality, Punctuality, Operation, and Support. The associations between these domains and variables such as age, education level, affected breast (unilateral or bilateral), type of oncologic surgery, performance of axillary lymphadenectomy, reconstruction moment (immediate or late) and type of reconstruction (with implants or autologous tissue) were analyzed.

In order to establish a parameter for comparison between the perception of patients and the expectation of hospital managers regarding the quality of the service, the authors held a meeting with the hospital’s management team, consisting of the Technical Director, the Administrative Director, the Nursing Director, the Coordinator of the Plastic Surgery Service, and the Executive Director of the Hospital Maintaining Foundation. In this meeting, the objective of the study, the methodology applied, and the HSQS, with its domains and scores, were presented to the management team. The management team was asked to establish a minimum satisfactory score for the breast reconstruction service.

### Statistical method

The sample size was calculated from the number of breast reconstructions performed by the breast reconstruction service in 2017 and 2018 (110 procedures). Considering a sampling error of 5% and a confidence level of 95%, a minimum sample of 86 patients was established.

The Statistical Package for the Social Science (SPSS) version 20.0 was used for data analysis. Nominal and ordinal variables are here described as percentages and absolute frequencies. Chi-square and Spearman’s rank correlation coefficient tests were used to verify the relationship between the variables age, education level, affected breast (unilateral or bilateral), type of oncologic surgery, performance of axillary lymphadenectomy, reconstruction moment and type of reconstruction and HSQS domains. For all tests, significance was set to 5%.

## Results

A total of 97 patients were assessed for eligibility. After signing the consent form, 7 patients withdrew from filling out the questionnaire. Thus, 90 patients were included in data analysis. Time interval between surgery and assessment ranged from 1 to 17 months (mean ± SD: 6.6 ± 5.5; median: 6.0 months).

Table 1 presents the clinical and demographic characteristics of the participants. Types of reconstruction were stratified into two categories: *with the use of implants*, which included tissue expander, prosthesis alone or prosthesis covered by a local flap or a latissimus dorsi flap; and *with the use of autologous tissue* only, including reconstructions with locoregional flaps or transverse rectus abdominis musculocutaneous flap. The management team reached the consensus that the minimum satisfactory score for the breast reconstruction service would be 8.0, on a scale from 0 to 10. The mean overall percentage quality score obtained was 93.3%. Only one domain of the HSQS, ‘Support’, had an average score lower than that considered satisfactory by the management team. All the other domains had higher scores. The domain that scored highest was ‘Qualification,’ followed by ‘Result’ (Table 2).

**Table 1:** Patients’ main clinical and demographic characteristics

	Variable	N	%
Age group (years)			
	≤ 39	20	22.2
	40 - 59	60	66.7
	≥ 60	10	11.1
Education level			
	Elementary school	45	50.0
	High school	33	36.7
	College	12	13.3
Affected breast			
	Unilateral	80	88.9
	Bilateral	10	11.1
Type of oncologic surgery			
	Sectorectomy/quadrantectomy	11	12.2
	Subcutaneous mastectomy	3	3.3
	Radical mastectomy	76	84.4
Axillary lymphadenectomy			
	Yes	43	47.8
	No	47	52.2
Reconstruction moment			
	Immediate	79	87.8
	Late	11	12.2
Type of reconstruction			
	With the use of implants	69	76.7
	With autologous tissue only	21	23.3

**Table 2:** HSQS scores by domains (minimum satisfactory score: 8.00).

	Domain	Range	Mean ± SD^a^	Median ± IQR^b^
Block 1	Quality assigned to the service	7.1–10.0	9.69 ± 0.6	10.0 ± 0.4
	Satisfaction with the service	6.2–10.0	9.70 ± 0.7	10.0 ± 0.3
	Intentions of loyalty to the service	7.9–10.0	9.82 ± 0.4	10.0 ± 0.0
Block 2	Quality interpersonal relationships	7.1–10.0	9.83 ± 0.4	10.0 ± 0.0
	Technical quality	4.7–10.0	9.18 ± 1.3	10.0 ± 1.5
	Environment quality	7.1–10.0	9.73 ± 0.6	10.0 ± 0.0
	Administrative quality	7.1–10.0	9.82 ± 0.6	10.0 ± 0.0
Block 3	Interaction	0.8–10.0	9.66 ± 1.4	10.0 ± 0.0
	Relationship	4.2–10.0	8.74 ± 1.6	9.5 ± 2.3
	Result	6.6–10.0	9.86 ± 0.4	10.0 ± 0.0
	Qualification	7.1 - 100	9.94 ± 0.3	10.0 ± 0.0
	Environment	2.5–10.0	8.80 ± 1.5	9.1 ± 2.0
	Aesthetics and functionality	3.0–10.0	8.77 ± 1.6	9.5 ± 1.9
	Punctuality	1.4–10.0	8.23 ± 2.3	9.2 ± 2.9
	Operation	5.9–10.0	9.58 ± 0.7	10.0 ± 0.5
	Support	1.4–10.0	7.22 ± 3.0	8.5 ± 4.3
	Overall quality score	1.4–10.0	9.33 ± 0.6	9.6 ± 0.7

a SD: standard deviation;

b IQR: Interquartile range.

There were weak correlations between patients’ variables and some domains of the HSQS. There was a weak positive correlation between ‘type of oncologic surgery’ and ‘intentions of loyalty to the service’ (ρ = 0.272; p = 0.009), meaning that patients who had undergone mastectomy intended to continue treatment in the service and would recommend it to other patients in the same situation. There was a weak negative correlation between ‘education’ and ‘quality of the environment (ρ = –0.218; p = 0.039), indicating that the higher the patient’s level of education, the lower they perceived the quality of the service’s environment.

It was also observed that the higher the patient’s level of education, the higher the score attributed to ‘relationship’ (ρ = 0.261; p = 0.013) and the lower the score attributed to ‘aesthetics and functionality’ (ρ = –0.237; p = 0.024).

When the scores of HSQS domains were stratified into ≥ 8.00 or < 8.00 (minimum score considered satisfactory for the service), significant associations were observed between the domain ‘relationship’ and two variables, ‘axillary lymphadenectomy’ and ‘moment of reconstruction’: 69.6% of patients who had undergone axillary lymphadenectomy scored the relationship with the team lower than 8.0 (p = 0.015), and 92.5% of patients who underwent immediate breast reconstruction scored relationship with the team higher than 8.0 (p = 0.019) (chi-square test). There was no other significant association between the studied variables and the domains of the HSQS.

## Discussion

In Brazil, health service managers consider the basis of quality to be the association between customer satisfaction, low risks and low rates of incidents related to the care provided^8^^,^ 9. Management of the health services quality can result in greater profitability through customer loyalty, cost reduction, and optimized use of available resources^10^^,^ 11.

Accreditation processes and certifications are essential to detect deficiencies and continuously improve quality of health institutions^9^^,^ 12. Accreditation occurs when the institutional reality is consistent with specific quality standards previously defined by the accreditation of a given country, as determined by periodic external evaluation. Brazilian government does not have a mandatory quality assessment program for health services. However, there are specific institutions around the world that perform accreditation, contributing to the implementation of best practices in the health sector, such as the National Accreditation Organization (NAO) in Brazil, the Joint Commission International, the Canadian Council on Healthcare Services Accreditation, and the National Integrated Accreditation for Healthcare Organizations^13^.

NAO’s Quality Seal Manual sets a minimum score of 80% of compliance^14^. Thus, the quality of the breast reconstruction service was considered high, reaching an overall HSQS score of 9.33. These positive results are consistent with the findings reported by Cohen et al.^5^, in which 1,534 patients who underwent breast reconstruction considered satisfactory the interaction with the plastic surgeon and the health teams, although opportunities for further improvement were observed concerning the information provided to them.

The customer’s perceptions of the quality of health services help the management team to make decisions regarding the allocation of financial and human resources. Therefore, it is important that the hospital’s management team, and not those responsible for the service, determine the minimum score considered satisfactory^7^.

In the present study, 87.8% of the participants had undergone immediate reconstruction, and the most used technique involved an expander/implant (76.7% of the procedures). From the surgeon’s perspective, immediate reconstruction is usually better. A recent study of plastic surgeons’ perspective on issues related to breast cancer treatment and reconstruction showed that 64.9% of surgeons reported recommending immediate reconstruction with an expander/implant^15^.

The concomitant axillary lymphadenectomy tends to negatively affect the patient’s relationship with the team: 69.6% of the women who underwent lymphadenectomy evaluated their relationship with the team under 8.00. These results corroborate the findings of Bregagnol and Dias^16^, who observed that patients who undergo axillary lymphadenectomy presented more complications, especially in the immediate postoperative period, and this may have compromised the relationship with the team.

The domain ‘Support’ was the only that scored < 8.00. It involves asking participants whether the service organizes groups or patient support programs, whether a wide variety of patient support services is available, and whether the service offers care that go beyond medical treatment. The score obtained indicates the need for greater investment in support networks offered to women undergoing breast reconstruction.

The changes arising from the treatment performed for breast cancer are mainly linked to physical appearance and psychosocial changes, such as signs of anxiety, decreased libido, and physical, social, and financial problems^17^^-^ 19. The psychosocial support offered by support groups is effective for adjusting and improving patients’ quality of life, and this may improve the ability of these women to adapt to life after surgery^18^^,^ 20.

Networking with other breast cancer patients can help women accept their condition. Patients planning to undergo breast reconstruction usually appreciate talking to other women who had undergone the procedure. Pestana^21^ observed that 66% of women reported that they would attend two or more meetings to learn about breast reconstruction.

There are Brazilian federal laws that guaranteed the right to breast reconstruction to all patients undergoing breast cancer treatment. However, the postoperative period after breast reconstruction needs attention. Support for breast cancer patients should consider the broader context of women’s lives, which includes psychological, cultural, educational, economic, and social factors. Consistent with these results for the domain ‘Support,’ a recent study observed that there is considerable fragility in the services and support networks available for breast cancer patients^22^.

Few instruments assess patient satisfaction regarding the quality of health services, and most of them evaluate services generically and superficially^23^. An analysis of quality measurement instruments demonstrated that generic instruments are not sufficient to assess the quality of health services and that developing countries should seek or even develop models that address their sociocultural and technical reality^24^.

This study has several limitations, including the small sample size and the wide time interval between surgery and patient’s assessment (1 to 24 months). A narrower time interval, with patients in more similar postoperative stages, would allow for greater sample homogeneity. Another limitation is the lack of data from other breast reconstruction services or even for other specific health care services in Brazil to compare with our results. Further, it is necessary to take into account the fact that data collection was performed before the COVID-19 pandemic. The context of social distancing, prevention, and safety measures may have changed the patients’ perceptions of the quality of the evaluated service.

This study is an initial step; much research on this topic is needed. However, a validated tool was used to assess a topic that has been little studied in Brazil’s public or philanthropic hospitals, and the knowledge of patients’ perception of the treatment offers valuable opportunities for the improvement of the service, inspiring the creation of support groups, multiprofessional care groups, and institutional policies to support patients undergoing breast reconstruction.

## Conclusion

According to the patients, the quality of the breast reconstruction service is satisfactory. However, the results of the current study highlighted a need for structural improvements, better interpersonal relationships, and a stronger support network for patients.

## Data Availability

All data sets were generated or analyzed in the current study.

## References

[1] [INCA] Instituto Nacional do Cancer (2022). Incidência: Apresenta dados de incidência do câncer de mama no Brasil, regiões e estados.

[2] Ortega CCF, Veiga DF, Camargo K, Juliano Y, Sabino M, Ferreira LM (2018). Breast reconstruction may improve work ability and productivity after breast cancer surgery. Ann Plast Surg.

[3] Archangelo SCV, Sabino M, Veiga DF, Garcia EB, Ferreira LM (2019). Sexuality, depression and body image after breast reconstruction. Clinics.

[4] Fontes KP, Veiga DF, Naldoni AC, Sabino-Neto M, Ferreira LM (2019). Physical activity, functional ability, and quality of life after breast cancer surgery. J Plast Reconstr Aesthet Surg.

[5] Cohen WA, Ballard TNS, Hamill JB, Kim HM, Chen X, Klassen A, Wilkins EG, Pusic AL. (2016). Understanding and optimizing the patient experience in breast reconstruction. Ann Plast Surg.

[6] Dagger TS, Sweeney JC, Johnson LW (2007). A hierarchical model of health service quality. J Serv Res.

[7] Rocha LR, Veiga DF, Oliveira PR, Song EH, Ferreira LM (2013). Health service quality scale: Brazilian Portuguese translation, reliability and validity. BMC Health Serv Res.

[8] Oliveira JLC, Gabriel CS, Fertonani HP, Matsuda LM (2017). Management changes resulting from hospital accreditation. Rev Lat Am Enfermagem.

[9] Corrêa JÉ, Turrioni JB, Paiva AP, Paes VC, Balestrassi PP, Papandrea PJ, Gonçalves EDC (2018). The influence of accreditation on the sustainability of organizations with the Brazilian accreditation methodology. J Healthc Eng.

[10] Gabriel CS, Bogarin DF, Mikael S, Cummings G, Bernardes A, Gutierrez L, Caldana G. (2017). Brazilian nurses’ perspective on the impact of hospital accreditation. Enferm Glob.

[11] Caldana G, Gabriel CS (2017). Evaluation of the hospital accreditation program: Face and content validation. Rev Bras Enferm.

[12] Oliveira JLC, Cervilheri AH, Haddad MCL, Magalhães AMM, Ribeiro MRR, Matsuda LM (2020). Interface between accreditation and patient safety: nursing team perspectives. Rev Esc Enferm USP.

[13] Berssaneti FT, Saut AM, Barakat MF, Calarge FA (2016). Is there any link between accreditation programs and the models of organizational excellence?. Rev Esc Enferm USP.

[14] Cruz PG (2021). Manual para organizações prestadoras de serviço de saúde – OPSS: roteiro de construção do manual brasileiro de acreditação ONA 2022.

[15] Momoh AO, Griffith KA, Hawley ST, Morrow M, Ward KC, Hamilton AS, Shumway D, Katz SJ, Jagsi R (2020). Postmastectomy breast reconstruction: Exploring plastic surgeon practice patterns and perspectives. Plast Reconstr Surg.

[16] Bregagnol RK, Dias AS. (2010). Alterações funcionais em mulheres submetidas à cirurgia de mama com linfadenectomia axilar total. Rev Bras Cancerol.

[17] Barrera I, Spiegel D. (2014). Review of psychotherapeutic interventions on depression in cancer patients and their impact on disease progression. Int Rev Psychiatry.

[18] Sousa H, Castro S, Abreu J, Pereira MG. (2019). A systematic review of factors affecting quality of life after postmastectomy breast reconstruction in women with breast cancer. Psychooncology.

[19] Cesnik VM, Vieira EMV, Giami A, Almeida AM, Santos DB, Santos MA (2013). The sexual life of women with breast cancer: Meanings attributed to the diagnosis and its impact on sexuality. Estud Psicol.

[20] Mundy LR, Rosenberger LH, Rushing CN, Atisha D, Pusic AL, Hollenbeck ST, Hyslop T, Hwang ES (2020). The evolution of breast satisfaction and well-being after breast cancer: A propensity-matched comparison to the norm. Plast Reconstr Surg.

[21] Pestana IA (2020). Patient-guided breast reconstruction education. Cureus.

[22] Vargas GS, Ferreira CLL, Vacht CL, Dornelles CS, Silveira VN, Pereira ADA (2020). Social support network of women with breast cancer. Rev Pesq Cuid Fundam Online.

[23] Gasquet I, Villeminot S, Estaquio C, Durieux P, Ravaud P, Falissard B (2004). Construction of a questionnaire measuring outpatients’ opinion of quality of hospital consultation departments. Health Qual Life Outcomes.

[24] Endeshaw B. (2021). Healthcare service quality-measurement models: a review. J Health Res.

